# Methotrexate-Lactoferrin Targeted Exemestane Cubosomes for Synergistic Breast Cancer Therapy

**DOI:** 10.3389/fchem.2022.847573

**Published:** 2022-03-22

**Authors:** Sarah Mokhtar, Sherine N. Khattab, Kadria A. Elkhodairy, Mohamed Teleb, Adnan A. Bekhit, Ahmed O. Elzoghby, Marwa A. Sallam

**Affiliations:** ^1^ Cancer Nanotechnology Research Laboratory (CNRL), Faculty of Pharmacy, Alexandria University, Alexandria, Egypt; ^2^ Department of Industrial Pharmacy, Faculty of Pharmacy, Alexandria University, Alexandria, Egypt; ^3^ Chemistry Department, Faculty of Science, Alexandria University, Alexandria, Egypt; ^4^ Department of Pharmaceutical Chemistry, Faculty of Pharmacy, Alexandria University, Alexandria, Egypt; ^5^ Pharmacy Program, Allied Health Department, College of Health and Sport Sciences, University of Bahrain, Al-Manamah, Bahrain

**Keywords:** cubosomes, exemestane, breast cancer, methotrexate, lactoferrin

## Abstract

While the treatment regimen of certain types of breast cancer involves a combination of hormonal therapy and chemotherapy, the outcomes are limited due to the difference in the pharmacokinetics of both treatment agents that hinders their simultaneous and selective delivery to the cancer cells. Herein, we report a hybrid carrier system for the simultaneous targeted delivery of aromatase inhibitor exemestane (EXE) and methotrexate (MTX). EXE was physically loaded within liquid crystalline nanoparticles (LCNPs), while MTX was chemically conjugated to lactoferrin (Lf) by carbodiimide reaction. The anionic EXE-loaded LCNPs were then coated by the cationic MTX–Lf conjugate *via* electrostatic interactions. The Lf-targeted dual drug-loaded LCNPs exhibited a particle size of 143.6 ± 3.24 nm with a polydispersity index of 0.180. It showed excellent drug loading with an EXE encapsulation efficiency of 95% and an MTX conjugation efficiency of 33.33%. EXE and MTX showed synergistic effect against the MCF-7 breast cancer cell line with a combination index (CI) of 0.342. Furthermore, the Lf-targeted dual drug-loaded LCNPs demonstrated superior synergistic cytotoxic activity with a combination index (CI) of 0.242 and a dose reduction index (DRI) of 34.14 and 4.7 for EXE and MTX, respectively. Cellular uptake studies demonstrated higher cellular uptake of Lf-targeted LCNPs into MCF-7 cancer cells than non-targeted LCNPs after 4 and 24 h. Collectively, the targeted dual drug-loaded LCNPs are a promising candidate offering combinational hormonal therapy/chemotherapy for breast cancer.

## 1 Introduction

Breast cancer has one of the greatest occurrence rates among the different types of cancer globally. It ranks second to lung cancer with 1.67 million new patients per year (Gaber et al., 2020). Estrogen-dependent breast cancer accounts for two-thirds of postmenopausal breast carcinoma and one-third of breast cancer in general (Valle et al., 2005).

Exemestane (EXE) is a type I aromatase inhibitor. It binds irreversibly as a false substrate to the aromatase enzyme, which leads to the enzyme inactivation; this is called suicide inhibition (Valle et al., 2005). EXE has a minimal effect against other adrenal hormones because it specifically targets aromatase enzyme, which acts only in the rate-limiting step of estrogen synthesis (Robinson, 2009). It is approved for hormone-dependent breast cancer as a first-line treatment (Elzoghby et al., 2017). Its structure is related to androstenedione found naturally in the body (Valle et al., 2005). It is very lipophilic due to its steroidal structure with a log *p* = 4.2 and a poor aqueous solubility of 80 μg*/*ml. Unfortunately, aromatase inhibitors may suffer failure of therapy in case of developed resistance. In case of EXE, one of the causes of the resistance could be its weak estrogenic property that can cause upregulation of AREG, which is an epidermal-like growth factor that activates epidermal growth factor receptor causing proliferation of cancer cells. The P-glycoprotein efflux pumps overexpression on cancerous cells can also cause resistance to EXE (Elzoghby et al., 2017).

To enhance the therapeutic efficacy of EXE, it should be combined with another agent that acts through a different signaling pathway to augment the antitumor efficacy while helping to reduce the dose and the associated adverse effects.

Methotrexate is an antimetabolite drug. It is a methyl derivative of aminopterin (Cipriani et al., 2014). It is considered a classical antifolate and, like most other antifolates, enters the cancerous cells mostly *via* reduced folate carrier (RFC) (Visentin et al., 2012). Since they are folate analogues, they act as competitors for folate in cellular uptake and intracellular polyglutamation. The MTX polyglutamate derivatives are vital for the cytotoxic action of MTX. They act as inhibitors to dihydrofolate reductase (DHFR) preventing formation of tetrahydrofolate, which is crucial in single carbon transfers for thymidylate, amino acids, and purine nucleotide synthesis. The reduced DHFR activity thus negatively affects the DNA synthesis (Howard et al., 2016; Karami et al., 2019). Inhibition of DHFR also prevents the activity of 5-aminoimidazole-4-carboxamide ribonucleotide (AICAR)-transformylase, which is important for *de novo* purine synthesis (Cipriani et al., 2014). Resistance against MTX could be to impairment of RFC, mutations of folate metabolic enzymes, or MDR efflux transporter overexpression (Depau et al., 2017).

In this work, we aim to provide a targeted system for the combined delivery of EXE and MTX for breast cancer cells. Nanoparticles (NPs) offer great merits in cancer treatment over conventional therapy (Ahamed et al., 2015; Ahamed et al., 2021c). They can be made in variable sizes (1–1,000 nm), using extremely diverse materials including lipids, polymers, natural materials, and inorganic compounds (Pelicano et al., 2006). A nanocarrier should carry the drug, while avoiding the immune system, thus increasing the drug lifetime in the circulation, till releasing it in the targeted tissues (Estanqueiro et al., 2015).

Liquid crystalline nanoparticles (LCNPs) have gained a significant amount of attention because of their great potential in drug delivery (Guo et al., 2010). Liquid crystals, which are called mesophases, exist as an intermediate phase of matter between solid crystalline phase and true liquid (Lancelot et al., 2014). Thus, LCNPs have the best of both, which include a flexible structure, enhanced colloidal stability, and self-assemble ability (Kim et al., 2015; Gabr et al., 2017). They also have prolonged release profile owing to their very ordered and organized internal structures. LCNPs show better structural and storage stability at room temperature and lesser drug leakage than liposomes and they can also withstand thermal treatment (Peng et al., 2014).

Cubic-phase LCNPs (cubosomes) are formed as stable colloidal dispersions, where the amphiphilic polar lipids self-assemble when introduced to a certain ratio of surfactant and water (Angelova et al., 2011). They have a microstructure that is composed of a curved bicontinuous lipid bilayer, extended in 3D, and two discrete interpenetrating aqueous channels, with a large interfacial area (Guo et al., 2010).This multicompartment model in the cubosomes makes them an excellent carrier for drugs of different solubility (lipophilic, hydrophilic, and amphiphilic) (Guo et al., 2010).

Various liquid crystal building amphiphilic lipids have been reported, with glyceryl monooleate or monolein being the most reported (Freag et al., 2016b). GMO is generally recognized as safe (GRAS) and is FDA approved as an inactive ingredient (Garg et al., 2007). GMO can peculiarly form cubic structures in a broad range of system composition and temperature (Lancelot et al., 2014).

Lactoferrin (Lf) is a glycoprotein, belonging to the transferrin family, which are primarily iron-transporting proteins (El-Hawy et al., 2021). It was reported that milk-derived Lf can decrease the viability of human ductal breast epithelial tumor T47D cells and breast cancer HS578T cells by 54% and 47%, respectively, and duplicate apoptosis rate in these cells (Duarte et al., 2011). Some overexpressed receptors on tumor cells such as Lf receptors (LRP1 and LRP2) and low-density lipoprotein can act like gates for Lf to enter cancer cells (Metawea et al., 2021). Moreover, nuclear localization sequence of Lf can help nucleus targeting of drugs attached to it (Baumrucker et al., 2006; Abdelmoneem et al., 2021). Lf cationic nature permits charge-based binding to anionic ligands like glycosaminoglycans on cell surface for cellular uptake and internalization (Agwa and Sabra, 2020; Ahamed et al., 2021a). Those properties attracted many scientists to use Lf as an active ligand to achieve targeted delivery of drugs to the cancerous cells. Moreover, the structural functionalities of LF enable its chemical conjugation to a variety of drugs forming polymer–drug conjugates. The latter have been employed as a strategy to enhance drug stability, solubility, and circulation time (Tran and Tran, 2019). Moreover, they help protect the normal body cells against the cytotoxic effect of chemotherapeutics by controlling the drug to be released at specific sites and in the presence of specific enzymes (Dolz-Pérez et al., 2020; Sallam et al., 2020). Additionally, Lf being a component of body innate immune system possesses merits compared to synthetic polymers that are employed for the synthesis of polymer–drug conjugates.

In this study, we propose for the first time, to our knowledge, the simultaneous codelivery of the aromatase inhibitor exemestane and the antimetabolite methotrexate by optimizing a targeted hybrid formulation encompassing lactoferrin–MTX conjugate enveloping EXE-loaded LCNPs. LCNPs were chosen for their high encapsulation efficiency of hydrophobic drugs and their highly negative charge that permits electrostatic interaction. The hydrophilic glycoprotein Lf was selected for its targeting action to the Lf receptors expressed on breast cancer cells, and for its ability of escaping the opsonization, hence prolonging the systemic circulation besides its immune-modulating action and its biodegradability. The proposed formulation is planned to be employed for breast cancer targeted therapy.

## 2 Experimental Section

### 2.1 Materials

The details of materials including chemicals and solvents were shown in the supporting information.

### 2.2 Preparation of Self-Assembled Liquid Crystalline Nanoparticles

#### 2.2.1 Synthesis of LCNPs F1

The method of Esposito et al. was followed for the preparation process of LCNPs, F1 with certain modification (Esposito et al., 2005). Briefly, 200 mg of glyceryl monooleate (GMO), representing the lipid phase, was melted at 60°C in a thermostatically controlled water bath. An aqueous phase consisting of 10 ml of poloxamer 407 (P407) solution (0.5% w/v) was prepared and heated to 60°C and then was gently added to the melted lipid followed by probe sonication at 70% amplitude for 10 min (Thapa et al., 2015; Elgindy et al., 2016). The final dispersion was cooled and stored at ambient temperature for further investigation. The LCNPs **F1** were then lyophilized using mannitol 5% w/v as a cryoprotectant for solid-state characterization.

#### 2.2.2 Physical Loading Technique of EXE Within LCNPs F2

EXE was physically loaded into the hydrophobic monoglyceride bilayer of LCNPs (Elgindy et al., 2016). Briefly, EXE (5% w/w) was solubilized in 200 mg of melted GMO at 60°C using a bath sonicator. An aqueous phase (0.5% w/w P407) at 60°C was slowly added to the melted lipid followed by probe sonication at 70% amplitude for 10 min. EXE-LCNPs **F2** were then lyophilized for further characterization. The encapsulation efficiency of the LCNP dispersions was quantified using HPLC.

#### 2.2.3 Preparation of Methotrexate–Lactoferrin Conjugate F3

Methotrexate (MTX, 0.005 g, 0.011 mmol) was dissolved in 3 ml (0.3 M ammonium acetate, pH 7). EDC.HCl (0.005 g, 0.028 mmol) and K salt of Oxyma (0.005 g, 0.028 mmol) were added to the MTX solution (Khattab, 2010; Subirós-Funosas et al., 2013; Jad et al., 2015). The solution was then preactivated at ambient temperature for 7–10 min under constant stirring. One hundred milligrams of lactoferrin was then dissolved in 2 ml (0.3 M) of ammonium acetate and added to the preactivated MTX solution to react with the MTX active ester. Triple coupling was performed, where, after 2 and 4 h, another 5 mg of MTX was preactivated by the equivalent amount of EDC.HCl and K-Oxyma and added to the reaction mixture. Subsequently, the reaction mixture was stirred at ambient temperature for 24 h and was then dialyzed against 0.3 M ammonium acetate solution for 24 h to reach equilibrium. The unreacted drug was determined from using dialysis medium. Dialysis of the reaction mixture was completed against distilled water for further purification. The resulting solution was then lyophilized for solid-state characterization.

#### 2.2.4 Optimization of Targeted Dual-Loaded LCNPs, (MTX/Lf\EXE\LCNPs) F4

Targeted dual-loaded LCNPs were simply prepared by electrostatic attraction of the anionic EXE-loaded LCNPs **F2** and the cationic MTX–Lf conjugate **F3**. Different amounts of MTX–Lf conjugate **F3** were slowly added to 5 ml (5 mg EXE) of previously prepared EXE-loaded LCNPs **F2** under mild magnetic stirring for 30 min at RT to deposit a layer of the MTX–Lf conjugate **F3** onto the surface of **F2**. The final formulation **F4** was lyophilized for solid-state characterization using mannitol 5% w/w as a cryoprotectant.

### 2.3 Physiochemical Characterization

Drug loading was studied *via* HPLC, FTIR, MALDI TOF/TOF, ^1^H-NMR, and DSC. The surface charge was determined using Malvern Zetasizer. Also, particle size was determined by means of Malvern Zetasizer, and TEM morphological analysis, physical stability (Fang et al., 2012), and solid-state characterization (Elgindy et al., 2011; Ali et al., 2020) were performed and detailed in the Supplementary Material.

### 2.4 *In vitro* Drug Release

*In vitro* release of EXE and MXT from self-assembled LCNPs **F2** and **F4** was compared to free drug suspension using the dialysis bag technique as explained in the Supplementary Material (El-Far et al., 2018a).

### 2.5 *In vitro* Serum Stability

The targeted dual drug-loaded LCNPs **F4** was tested for its *in vitro* serum stability as mentioned in the Supplementary Material (Wolfram et al., 2014; El-Far et al., 2018b).

### 2.6 *In vitro* Cytotoxicity Study and Cellular Uptake

*In vitro* cytotoxicity of EXE, MTX, their combination, and the MTX/Lf\EXE\LCNPs **F4** on the MCF-7 breast cancer cell line was examined using the MTT assay. The evaluation of the cellular uptake of free coumarin-6, coumarin-6-labeled non-targeted LCNPs, and Lf-targeted LCNPs into MCF-7 breast cancer cells was done by using confocal microscopy and flow cytometry as described in the Supplementary Material.

### 2.7 Statistics

For all *in vitro* characterization, all tests were done in triplicate and values are presented as the average ± S.D. For comparison of mean values between groups, paired *t*-test, analysis of variance (ANOVA) test, and Tukey’s multiple comparison test were used. The difference was considered significant when *p*-values <0.05.

## 3 Results and Discussion

### 3.1 Synthesis of LCNPs F1

Emulsification technique was employed to fabricate LCNPs using GMO, P407, and water at 60°C with modification. GMO is made of the glycerol moiety that represents the polar head and the lipophilic tail revealed by the C_18_ hydrocarbon chains (Sagalowicz et al., 2006). The hydrophobic chains tend to melt easily at a higher temperature while the polar head remained intact and strongly bounded (Sagalowicz et al., 2006). The structural assembly of highly ordered atoms and melted ones were the main particularity of cubosomal structure (Abdelrahman et al., 2015). However, GMO itself does not form a stable emulsion in water and requires an emulsifier. It has been reported that poloxamer extremely increases the stability of the vesicle state occurring in lipid dispersions. In particular, P407 was shown to effectively stabilize dispersions of hexagonal and bicontinuous cubic phases. The phase diagram of monoolein/P407 shows that the surfactant is not just absorbed at the particle surface. It is thought that the polyethylene oxide (PEO) tails are solubilized in water, while the polypropylene oxide (PPO) blocks of P407 are anchored in the non-polar region or at the surface of the monoglyceride-based bilayers. This disposition should stabilize the vesicles toward fusion by a strong steric repulsion between bilayers (Esposito et al., 2003). P407 can interfere with the GMO molecules resulting in disruption in membrane lipid forming the cubic phase (Chong et al., 2011). Thus, both GMO and P407 contribute in forming highly ordered cubic nanoparticles. GMO was applied in 2% w/v concentration and P407 in 0.5% w/v concentration, which was reported by Abdelaziz et al. and Freag et al. to give the optimum particle size, PDI, and zeta potential to stabilize the LCNP dispersion and preserve its inner structure (Freag et al., 2016a; Elgindy et al., 2016; Abdelaziz et al., 2019).

### 3.2 Physical Loading Technique of EXE Loading Within LCNPs; EXE/LCNPs F2

**SCHEME 1:**
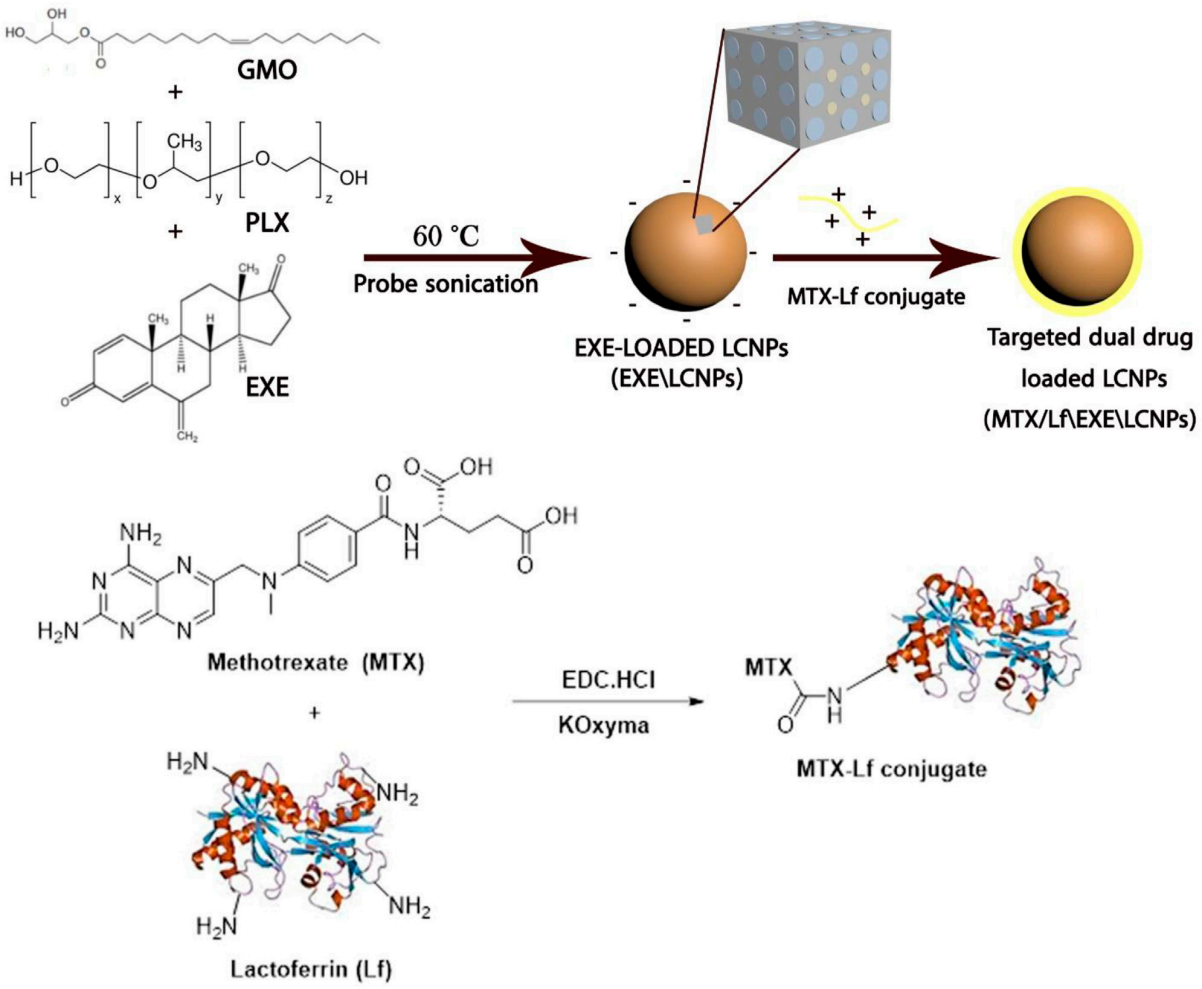
Schematic presentation of the preparation of targeted dual drug-loaded LCNPs, MTX/Lf\EXE\LCNPs **F4**.

To lower the dose of the aromatase inhibitor EXE and decrease its associated adverse effects, it was successfully loaded within LCNPs at 1% w/v with regard to the final LCNPs dispersion (Scheme 1). The distinctive absorption bands of EXE on the HPLC analysis proved that it was successfully loaded into LCNPs. Loading of EXE has negligible effect on physicochemical properties of LCNPs, since EXE-loaded LCNPs **F2** have almost similar PS, PDI, and zeta potential to those of the blank LCNPs **F1** (Table 1). A high encapsulation efficiency (95%) of EXE in the LCNPs was obtained, which could be attributed to the strong affinity of the hydrophobic EXE to the hydrophobic monoglyceride bilayer allowing almost complete incorporation with negligible drug amount released out to the dispersion medium. A similar behavior has been previously reported for progesterone in the GMO-based cubic nanoparticles that showed an EE in the range of 94.2%–99% (Elgindy et al., 2016).

**TABLE 1:** Physicochemical characterization, particle size, zeta potential, encapsulation efficiency (EE), conjugation efficiency (CE), and drug loading (DL) of blank and drug-loaded LCNPs (*n* = 3).

	Formulation	Initial drug loading		Particle size (nm)	Zeta potential (mV)	PDI	EXE		MTX	
		EXE (mg)	MTX (mg)				EE wt.%	DL wt.%	CE wt.%	DL wt.%
F1	Blank LCNPs	—	—	124 ± 4.57	−28.5	0.145	—	—	—	—
F2	EXE/LCNPs	10 mg	—	137.3 ± 4.06	−28.4	0.184	95%	3.66%	—	—
F3	MTX–Lf conjugate	—	15 mg	—	—	—	—	—	33.33%	4.76%
F4	MTX/Lf\EXE\LCNPs	5 mg	15 mg	143.6 ± 3.24	+5.64	0.180	95%	2.02%	33.33%	2.13%

### 3.3 Synthesis of MTX–Lf Conjugate F3

**FIGURE 1:**
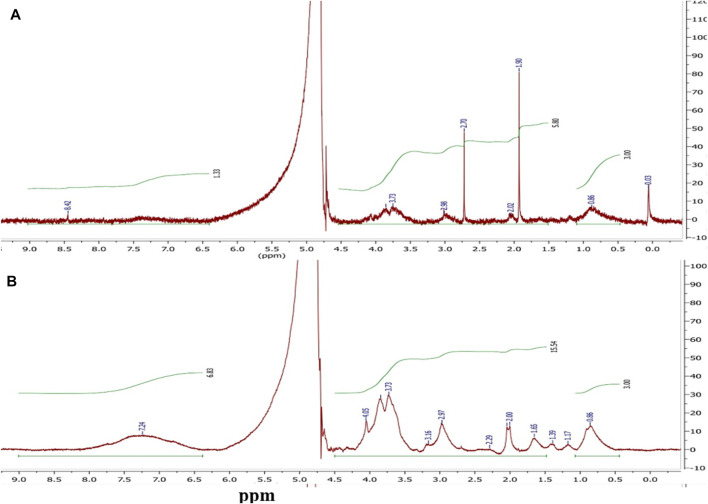
^1^H-NMR (D_2_O, 500 MHz) spectra of **(A)** Lf and **(B)** MTX–Lf conjugate **F3**.

Polymer–drug conjugation is a well-established and a widely applied technique to enhance the therapeutic properties of drugs. Polymer–drug conjugates typically show prolonged half-life, improved water solubility, and higher stability. Moreover, they have the advantages of improved permeability, site-specific drug release and drug retention in the cells (Swain et al., 2016; Anwar et al., 2018). Herein, MTX was conjugated to Lf backbone by an amide bond *via* simple carbodiimide coupling to prevent its release into systemic circulation and permit its release in tumor cells upon cleavage of the amide bond by lysosomal enzymes. MTX carboxylate groups were activated by using EDC. HCl and potassium salt of Oxyma forming the corresponding active ester (Cherkupally et al., 2013; Jad et al., 2015), which was allowed to react with the free amino groups of Lf to create an amide bond (Scheme 1). MTX–Lf conjugate could minimize the side effects, augmenting MTX accumulation within cancer cells and thus maximize its anti-tumor efficacy (Anwar et al., 2018; Sallam et al., 2020). Stehle et al. previously reported the synthesis of the MTX–HSA conjugate by direct carbodiimide coupling of MTX to the lysine residue in albumin (Stehle et al., 1997). The conjugation efficiency (CE %) of MTX to Lf backbone was calculated indirectly by quantifying the unconjugated (free) drug during equilibrium dialysis method using HPLC, where the conjugation percentage of MTX was about 33.33% (Table 1). The conjugation of the carboxyl group of MTX to the amino group of Lf was proved by FTIR,^1^H-NMR, DSC, and MALDI-TOF-MS. The ^1^H-NMR spectrum in D_2_O (Figure 1A) of Lf demonstrates several multiplet peaks at the range 0.50–4.20 ppm, which match the aliphatic protons of the peptide chain of Lf. Moreover, a broad multiplet (m) peak and a singlet peak at 7.00–7.60 and 8.42 ppm, respectively, which match the aromatic protons present in the peptide chain, are observed. The ^1^H-NMR spectrum of MTX–Lf conjugate **F3** (Figure 1B) shows a multiplet peak at the range of 0.50–1.00 ppm that corresponds to Lf. In addition, several multiplet peaks were detected at 0.50–4.20 ppm that match the aliphatic CH_2_ protons and N-CH_3_ protons of MTX [24], which are overlapped by the Lf aliphatic protons. Comparing the ^1^H-NMR spectra of both Lf and Lf-MTX (Figures 1A,B), one can observe an increase in the proton integration at 1.00–4.20 ppm, which confirms the successful conjugation of MTX and Lf. Additionally, the integration of protons at 7.00–8.50 ppm was remarkably increased, which correlates with the aromatic protons of MTX that overlap the Lf multiplet peak.

**FIGURE 2:**
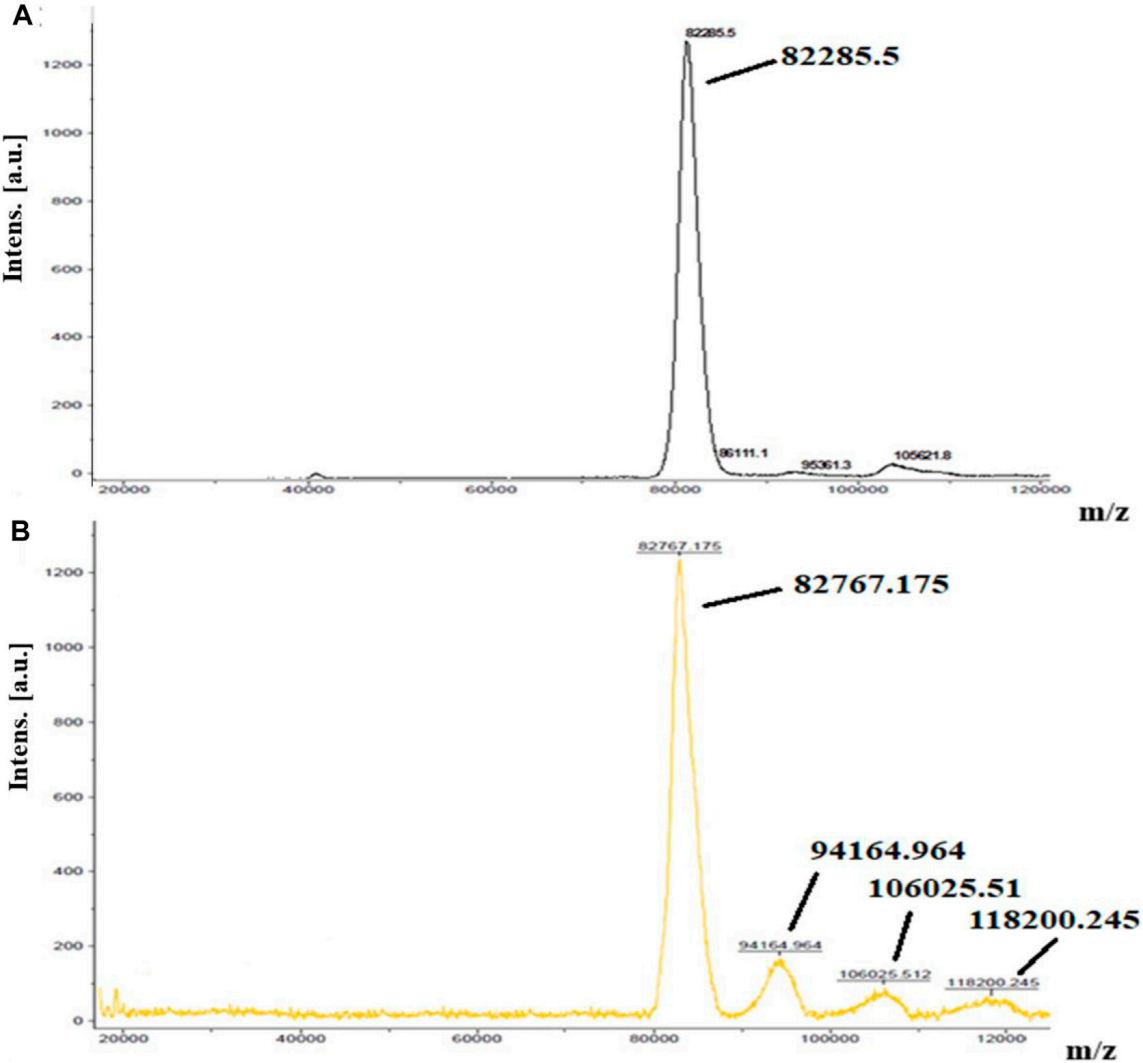
MALDI TOF mass spectra of **(A)** Lf and **(B)** MTX–Lf conjugate **F3**.

MALDI-TOF-MS was used to prove conjugation of Lf with MTX. One Lf molecule (molecular weight = 82,285.5 Da, Figure 2A) was linked to almost nine molecules of MTX resulting in Lf-MTX **F3** conjugate (molecular weight = 82,767.18, 94,164.97, 106,025.51, and 118,200.25, Figure 2B), equivalent to 4.8 mg MTX (DL% = 4.58%), which is in accordance with the DL% determined indirectly by HPLC (DL% = 4.76%, Table 1).

### 3.4 Optimization of Targeted Dual Drug-Loaded LCNPs, (MTX/Lf\EXE\LCNPs) F4

**FIGURE 3:**
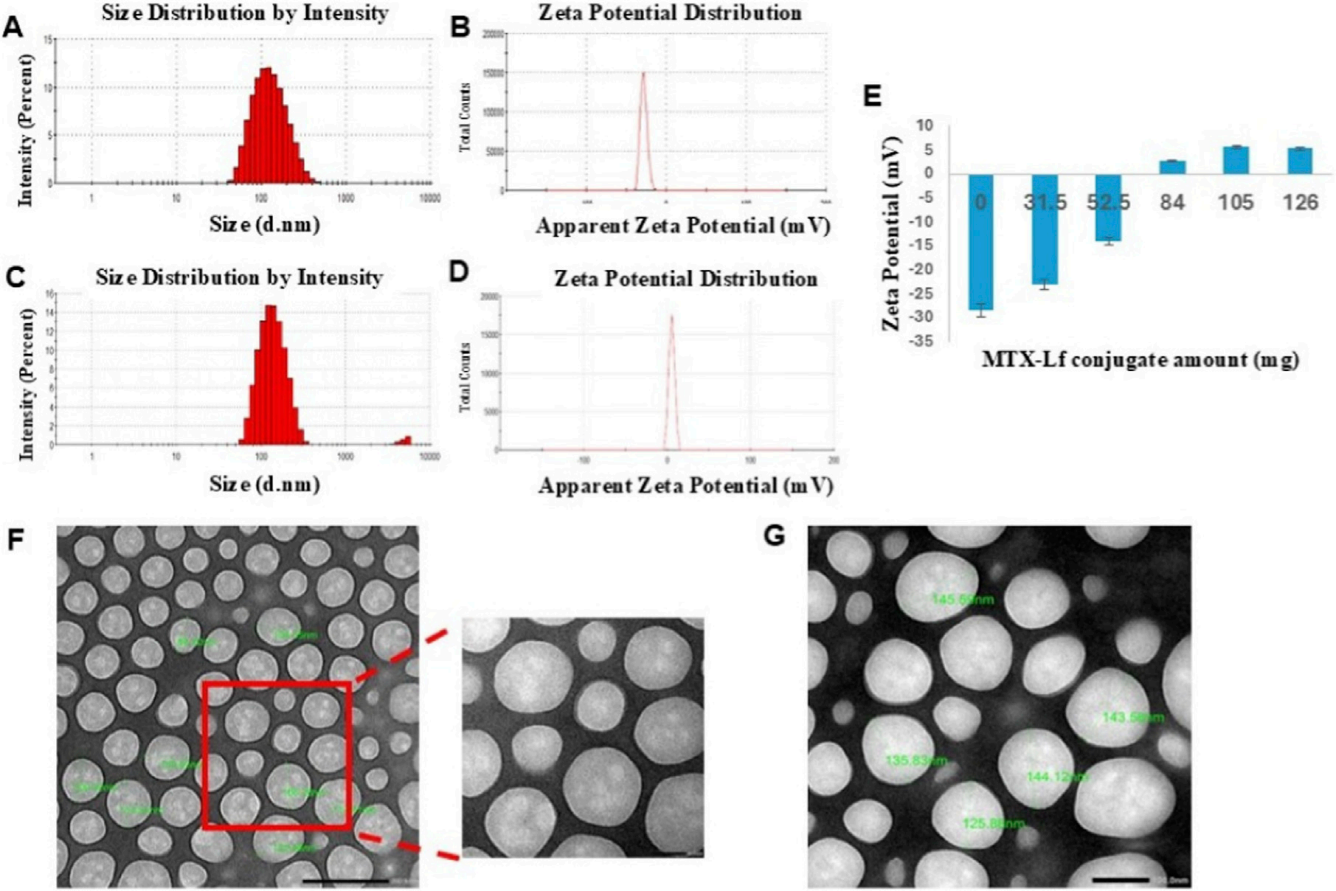
**(A)** Size distribution of EXE\LCNPs **F2**, **(B)** zeta potential of EXE\LCNPs **F2**, **(C)** size distribution of MTX/Lf\EXE\LCNPs **F4**, **(D)** zeta potential of MTX/ Lf\EXE\LCNPs **F4**, **(E)** effect of addition of MTX -Lf conjugate **F3** amounts on the zeta potential of EXE\LCNPs **F2**, **(F)** TEM of EXE\LCNPs **F2**, and (G) TEM of MTX/ Lf\EXE\LCNPs **F4**.

One of the prominent strategies used to enhance the stability and prolong the NPs systemic circulation is the modification in the nanocarrier composition. Hydrophilic polymers are employed for covering nanoparticles’ surface to sustain the systemic circulation and to adequately target tumor cells. They do so by repelling plasma proteins and escaping the opsonization and elimination, which is termed the “cloud” effect (Swain et al., 2016). Natural polymers including polysaccharides and proteins have been largely applied for active tumor targeting of nanocarriers by the virtue of their safety and their targeting to the overexpressed receptors on tumor cells (Abdelhamid et al., 2018). In our study, Lf was employed as a targeting ligand to improve LCNP internalization into breast cancer cells *via* binding to Lf receptors expressed on MCF-7 cells (Ali et al., 2020). Lf receptor-targeting mechanisms and charge-based interaction were exploited by electrostatic deposition of a positively charged layer of MTX–Lf conjugate onto negatively charged LCNPs. Different amounts of cationic MTX–Lf conjugate **F3** were added to 5 ml (equiv. to 5 mg EXE) of anionic EXE-loaded LCNPs **F2** to deposit a layer of the MTX–Lf conjugate on the LCNPs surface (Scheme 1). As the load of MTX–Lf conjugate **F3** was increased, the surface coverage was enhanced. Complete coverage was detected at the zeta potential charge reversal of EXE-loaded LCNPs from negative charge (−28.4 mV) into positive charge (+5.64 mV) for MTX/Lf\EXE\LCNPs **F4** at 105 mg of MTX–Lf conjugate (equiv. to 5 mg MTX) (Figures 3B,D,E). Moreover, no significant increase in zeta potential value was found with further increase of MTX–Lf conjugate amount (Figure 3E). In addition, **F4** (143.6 ± 3.24 nm, Table 1, Figure 3C) showed larger particle size than EXE-loaded LCNPs (137.3 ± 4.06 nm, Table 1, Figure 3A), indicating the formation of an additional layer on the LCNP surface (El-Lakany et al., 2018).

### 3.5 Physiochemical Characterization of the Drug-Loaded Nano-Formulations

#### 3.5.1 Morphological Analysis

The particle size of nanoparticles is a major determining factor of their biological fate, with an optimum particle size of approximately 100–250 nm to avoid rapid elimination from the circulation (Ali et al., 2020). Both EXE\LCNPs **F2** and MTX/Lf\EXE\LCNPs **F4** showed spherical particles with a smooth surface. EXE\LCNPs **F2** had a range of particle size of approximately 100–122 nm, which are slightly smaller than the measurements acquired from DLS. The MTX/Lf\EXE\LCNPs **F4** showed a scope of particle size of 125–145 nm, which agrees with the measurements acquired from DLS. Moreover, lack of aggregation can be very well seen under TEM, indicating their excellent colloidal stability (Figures 3F,G).

#### 3.5.2 Fourier Transform Infrared Spectroscopy

**FIGURE 4:**
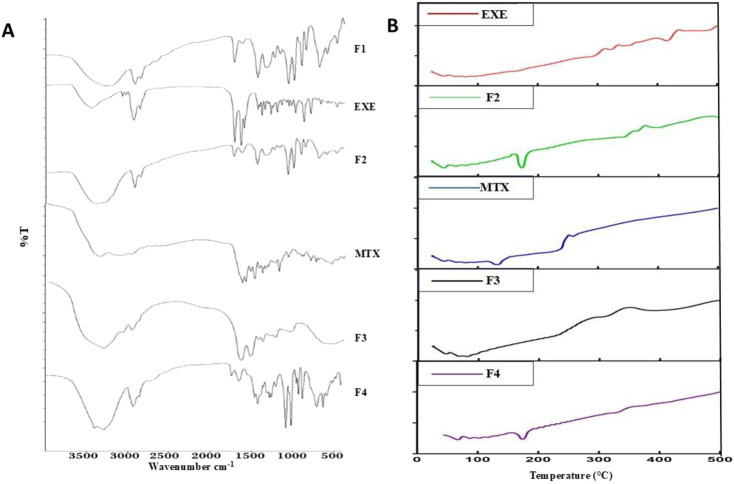
**(A)** FTIR spectra of blank LCNPs **F1**, EXE, EXE\LCNPs **F2**, MTX, MTX–Lf conjugate **F3**, and MTX/Lf\EXE\LCNPs **F4**. **(B)** DSC thermograms of EXE, EXE\LCNPs **F2**, MTX, MTX–Lf conjugate **F3**, and MTX/Lf\EXE\LCNPs **F4**.

Loading of EXE into LCNPs was proved by observing its characteristic bands at 1,737 cm^−1^ and 1,645 cm^−1^ corresponding to C=O stretching and C=C stretching bands, respectively. C–H stretching of EXE at 2,940 cm^−1^ overlapped with C–H stretching bands of P407 at 2,930 cm^−1^ (Figure 4A). FTIR spectrum of MTX–Lf conjugate **F3** indicates the disappearance of the characteristic peak of the carboxylic C=O group of MTX at 1,676 cm^−1^ confirming its conjugation with Lf. The stretching vibration band at 1,663 cm^−1^ that correlates with the new amidic carbonyl group in the conjugate overlaps the (amide I) distinctive band of Lf. The spectrum of MTX–Lf conjugate **F3** also shows broad stretching bands at the 3,600–2,500 cm^−1^ range, which fits the N–H and OH groups of Lf overlapped with those of MTX. The MTX/Lf\EXE\LCNPs **F4** FTIR spectrum showed what seems like a combined FTIR spectrum of that of EXE-loaded LCNPs and MTX–Lf conjugate. The broad band at 3,500–2,600 cm^−1^ reflects bands of Lf. The sharp band at 2,937 matches the sp3 C–H stretching band of EXE. The sharp band at 1740 cm^−1^ confirms the existence of EXE. The band at 1,663 cm^−1^ corresponds to the amidic carbonyl group of MTX–Lf conjugate **F3** (Figure 4A).

#### 3.5.3 Differential Scanning Calorimetry

DSC is an analytical technique that measures thermal properties (temperatures and heat flows) of a given formulation in relation to any transition in it as a function of temperature and time under a controlled atmosphere. The DSC thermogram of EXE-loaded LCNPs **F2** showed the disappearance of the endothermic peak of EXE at 85.6°C, confirming the entrapment of EXE and suggesting that entrapped EXE was molecularly dispersed in an amorphous state into the GMO (Figure 4B) (Elhasany et al., 2020). Moreover, disappearance of the MTX endothermic characteristic peak at 123.7°C in the thermogram of MTX–Lf conjugate **F3** indicates the successful conjugation between MTX and Lf (Figure 4B) (Kamel et al., 2019). On the other hand, the DSC thermogram of MTX/Lf\EXE\LCNPs **F4** revealed the absence of the endothermic peaks of EXE-loaded LCNPs **F2** observed at 61.9 and 350.0°C and the endothermic peaks of MTX–Lf conjugate **F3** observed at 79.1, 220.0, and 400°C.

#### 3.5.4 *In vitro* Drug Release

**FIGURE 5:**
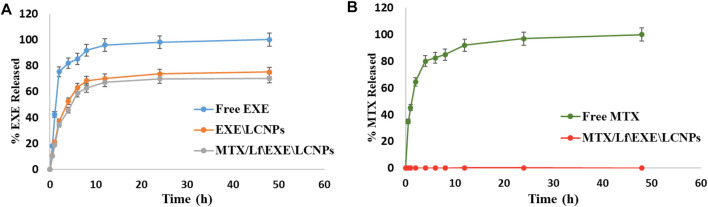
**(A)** *In vitro* release study of EXE from free EXE, EXE\LCNPs **F2**, and MTX/Lf\EXE\LCNPs **F4** at physiological pH 7.4 (*n* = 3). **(B)** *In vitro* release study of MTX from free MTX and MTX/Lf\EXE\LCNPs **F4** at physiological pH 7.4 (*n* = 3).

*In vitro* release of EXE showed a biphasic release pattern from both EXE\LCNPs **F2** and MTX/Lf\EXE\LCNPs **F4** formulations at physiological pH 7.4 (Figure 5A). At the first 2 h, about 35% of EXE was released from both **F2** and **F4** formulations. This relatively quick release was followed by a sustained EXE release of about 75% and 70% from **F2** and **F4**, respectively, over 48 h. In comparison, free EXE showed 85% drug release after only 6 h. Previous studies showed comparable outcomes where approximately 70% of EXE was released from nanocapsules over the period of 48 h (Gaber et al., 2019). The difference in *in vitro* release of EXE between the free EXE and both formulations (**F2** and **F4**) was statistically significant (*p*-value <0.001). The noticed initial fast release of EXE from the formulations **F2** and **F4** could be attributed to the dissociation of the drug adsorbed on the NP surface, where the high surface area-to-volume ratio of the LCNPs could be the reason for the observed initial fast release pattern. Nevertheless, the sustained release form of EXE obtained after the initial fast release period could be related to the diffusion of the lipophilic drug from the lipid compartments through the channels of the nano liquid crystalline matrix (Freag et al., 2016a; Mansour et al., 2017).

Regarding the release of MTX, almost all of the free drug was released (82.6%) after 6 h at physiological pH (Figure 5B). However, MTX conjugated to Lf demonstrated no drug release out of the final formulation **F4** over the entire window of *in vitro* release test (Figure 5B). This indicates the stability of the amide bond in MTX–Lf conjugate **F3** in the physiological pH. The difference in MTX *in vitro* release between the free MTX and **F4** was statistically significant (*p*-value <0.001). According to these findings, it is expected that this direct conjugation between MTX and Lf will restrict drug release in the circulation following parenteral administration resulting in minimal MTX concentration and thereby lowering its side effects. Meanwhile, lysosomal enzymes, at the tumor site, are expected to cleave the amide bond and release MTX (Zayed et al., 2019). This sustained release of the drug is favorable because it tends to reduce dosage frequency and toxicity (Ray et al., 2015).

#### 3.5.5 Physical Stability

**FIGURE 6:**
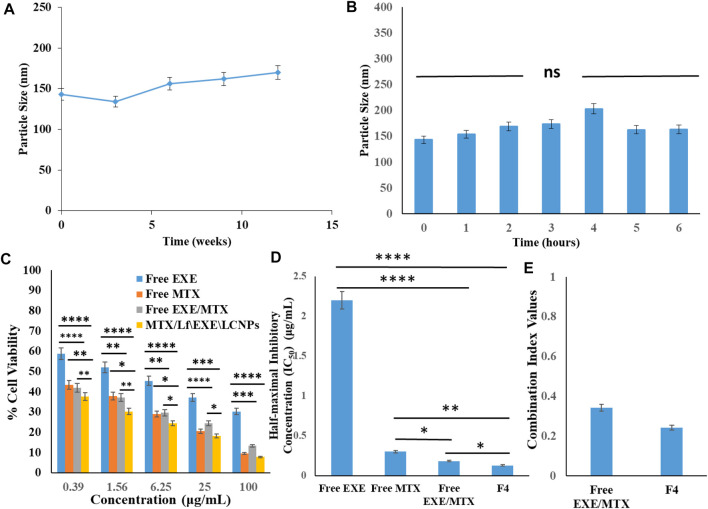
**(A)** Physical stability of MTX/Lf\EXE\LCNPs **F4** showing the change in particle size with time (3 months) (*n* = 3). **(B)** *In vitro* serum stability of MTX/Lf\EXE\LCNPs **F4** in 10% fetal bovine serum (FBS) solution according to PS measurements at different time intervals using DLS (*n* = 3). **(C)** Cytotoxicity analysis of free EXE, free MTX, and free EXE/MTX compared to targeted MTX/Lf\EXE\LCNPs **F4** on MCF-7 breast cancer cell line after 24 h (*n* = 3). **(D)** IC_50_ of free EXE, free MTX, EXE/MTX combination, and MTX/Lf\EXE\LCNPs **F4** (*n* = 3). **(E)** Combination Index (CI) of free drugs combination and MTX/Lf\EXE\LCNPs **F4** (*n* = 3). (ns, non-significant; *p < 0.05; **p < 0.01; ***p < 0.001; ****p < 0.0001).

After 3 months of storage at 4 ± 1°C, the prepared MTX/Lf\EXE\LCNPs **F4** demonstrated a particle size of 170 ± 4.16 nm and a zeta potential of +3.5 mV compared to the initial particle size 143.6 ± 3.24 nm and zeta potential of +5.64 mV (Figure 6A). The cationic MTX–Lf conjugate shell, even though it is of low positive charge, could have rendered both charge-based and steric stabilization. The latter is imparted by the glycol moiety of the Lf glycoprotein chain, which hinders the LCNPs’ coalescence (Abdelhamid et al., 2018; Abdelmoneem et al., 2019).

### 3.6 *In vitro* Serum Stability

Testing the *in vitro* serum stability of NPs is necessary for any formulation intended for parenteral administration, as unstable nanoparticles tend to interact with proteins forming aggregates (Aguilar-Castillo et al., 2015). Thus, the *in vitro* serum stability was tested to ensure the feasibility of parenteral administration. After 2 h of incubation, the size of the MTX/Lf\EXE\LCNPs **F4** increased from 143.6 ± 3.24 nm to 169 ± 2.58 nm probably because of serum proteins binding to the NP surface where they develop a protein corona (Ahamed et al., 2017) (Figure 6B). After 4 h, it reached 203.58 ± 2.23 nm, and this could be attributed to further serum protein accumulation on the surface of the NPs. After 6 h, the size decreased and reached 163.62 ± 3.01 nm (Figure 6B) due to serum protein dissociation off the surface of the nanoparticles (Abdelmoneem et al., 2019). Size shrinkage could also be due to the action of osmotic pressure by serum proteins. The osmotic action forces water outside the aqueous core causing shrinkage of the NPs (Freag et al., 2016a; Abdelmoneem et al., 2019). The PDI of the NPs ranged from 0.1 to 0.324 during the test period, which indicates high stability (Zayed et al., 2019).

### 3.7 *In vitro* Cytotoxicity Study and *in vitro* Cellular Uptake

#### 3.7.1 Cytotoxicity Assay

Cytotoxicity testing of free EXE, free MTX, free EXE/MTX combination, and the MTX/Lf\EXE\LCNPs **F4** was performed on MCF-7 breast cancer cells at 24 h using MTT assay (Ahamed et al., 2019). The IC_50_ of free EXE and free MTX were found to be 2.20 ± 0.059 μg/ml and 0.302 ± 0.005 μg/ml, respectively. The combined free EXE/MTX solution (1:1) showed 12- and 1.66-fold lower IC_50_ than that of EXE and MTX respectively, which indicates the synergistic cytotoxic action of this drug combination (Ahamed et al., 2021b). The MTX/Lf\EXE\LCNPs **F4** gave a lower IC_50_ value (0.128 μg/ml) than the free drug combination (Figures 6C,D). CompuSyn software (version 1) designed by Chou and Talalay was used to carry out statistical analysis. The combination index (CI) was analyzed to depict the synergism, additive effect, or antagonism. The CI of the combination of free drugs and MTX/Lf\EXE\LCNPs **F4** was 0.342 and 0.242, respectively. These values indicate synergistic cytotoxic action. Furthermore, the dose reduction index (DRI) of EXE and MTX was 34.14 and 4.7, respectively (Figure 6E).

#### 3.7.2 Confocal Microscopy Study

Confocal laser scanning microscopy was employed to visualize the cellular uptake efficiency of non-targeted LCNPs, Lf-targeted LCNPs, and free coumarin-6 dye by MCF-7 breast cancer cells. The MCF-7 cells were incubated with the formulations for 4 and 24 h. Coumarin-6 has been selected as a model hydrophobic fluorescent dye because it could be readily entrapped within the lipid bilayer of LCNPs.

**FIGURE 7:**
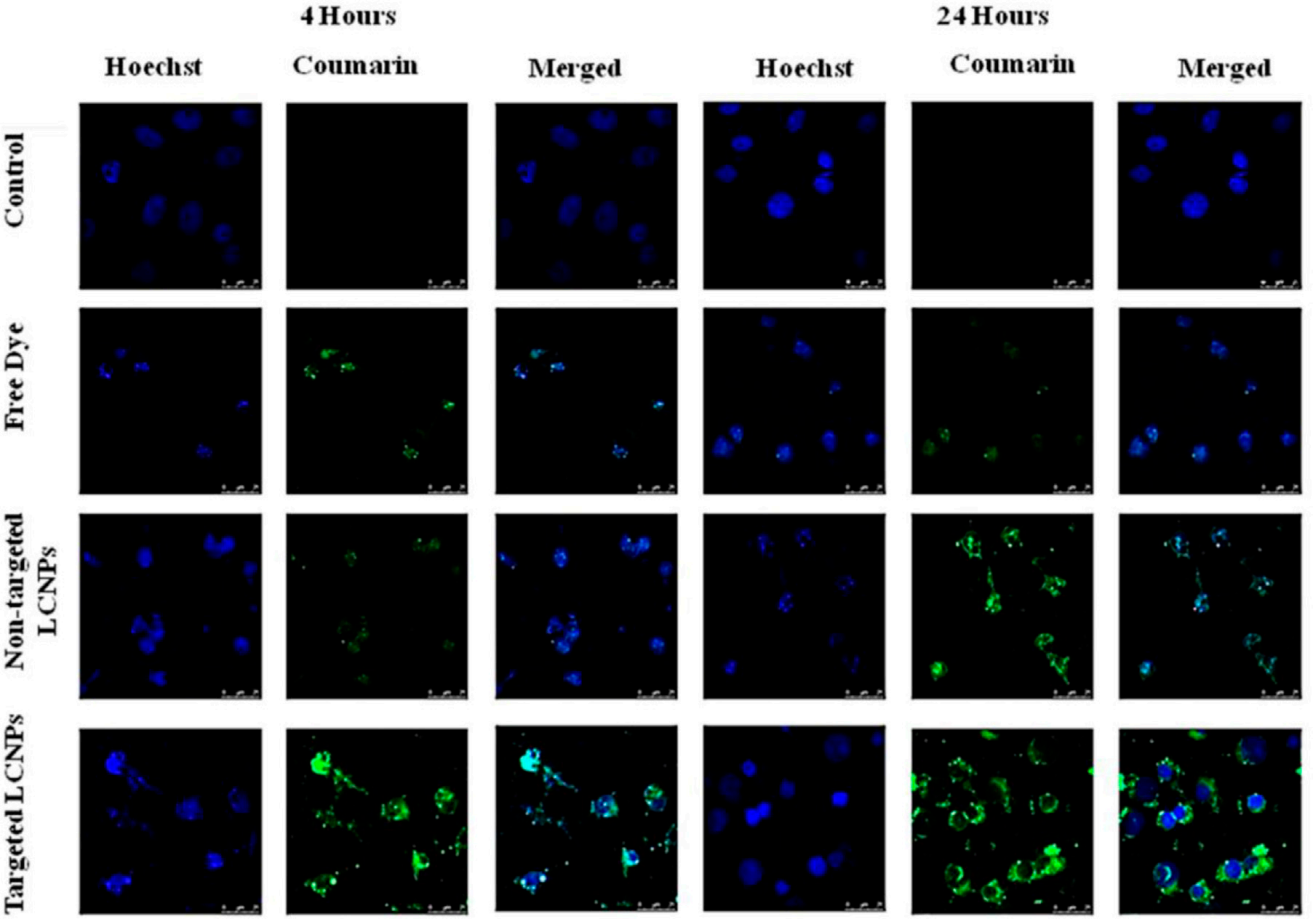
Confocal images showing cellular uptake of free coumarin dye, non-targeted coumarin-labeled LCNPs, and Lf-targeted coumarin-labeled LCNPs within MCF-7 breast cancer cells after incubation for 4 and 24 h.

The fluorescence images manifested that the LCNPs were distributed in the perinuclear region. Non-targeted NPs clearly demonstrated lower cellular uptake efficiency compared to Lf-targeted ones as suggested by the high green fluorescence intensity observed in cells treated with the latter. Conversely, the lowest green fluorescence intensity was seen in the cells treated with free coumarin-6 dye (Figure 7). After 24-h incubation, the intensity of fluorescence for the non-targeted and targeted LCNPs increased, confirming the time-dependent LCNP cellular uptake, while free coumarin-6 dye showed the weakest intensity of fluorescence suggesting low cellular uptake of free dye (Figure 7). Nanoparticle composition, size, and surface charge may direct the NP interaction with cellular membrane. The improved cellular uptake of coumarin-6-loaded LCNPs could be linked to GMO bio-adhesive and membrane fusing characteristics. Also, the peculiar structure of LCNPs, where the bicontinuous lipid bilayer surrounds water channels, offers a hydrophilic–hydrophobic pattern that helps interaction with the lipophilic cholesterol on cell surface (Abdelaziz et al., 2019). Moreover, enhanced NP cellular uptake could be expected to be due to their PS range of approximately 120–150 nm, since it has been well established that tumor vessels are extremely disorganized and dilated with many fenestrations between endothelial cells (100–600 nm). These physio-pathological characteristics of tumor vessels passively allow NPs to leak through tumor vasculature *via* the EPR effect and build up in tumor cells (Egusquiaguirre et al., 2012). In addition to the EPR effect of LCNPs, active targeting was done by functionalization of LCNPs with Lf, which allows the binding with Lf receptors over-expressed on the surface of tumor cells and augments cellular uptake *via* receptor-mediated endocytosis (RME).Moreover, cationic peptides have been found to act as endosomolytic agents in numerous formulations. Hence, being a cationic polymer, Lf can be an important factor for endosomal escape of LCNPs (Abdelhamid et al., 2018). Additionally, the positively charged surface imparted by the cationic Lf layer could further help the targeted LCNP cellular uptake by attaching to the anionic surface proteoglycans by adsorptive-mediated transcytosis (Abdelhamid et al., 2018; Abdelmoneem et al., 2019). On the other hand, the free dye could have entered the tumor cell *via* simple diffusion and encountered rapid saturation of intracellular region preventing further cellular uptake of free dye with time unlike NPs, which entered the tumor cells *via* endocytosis, which gave a gradual release of free dye from the NPs, hence avoiding intracellular saturation (Abdelaziz et al., 2019). Lf-targeted mesoporous silica NPs (MSNPs) were reported to show better cellular uptake when compared to untargeted MSNPs (Ali et al., 2020).

#### 3.7.3 Flow Cytometry Study

**FIGURE 8:**
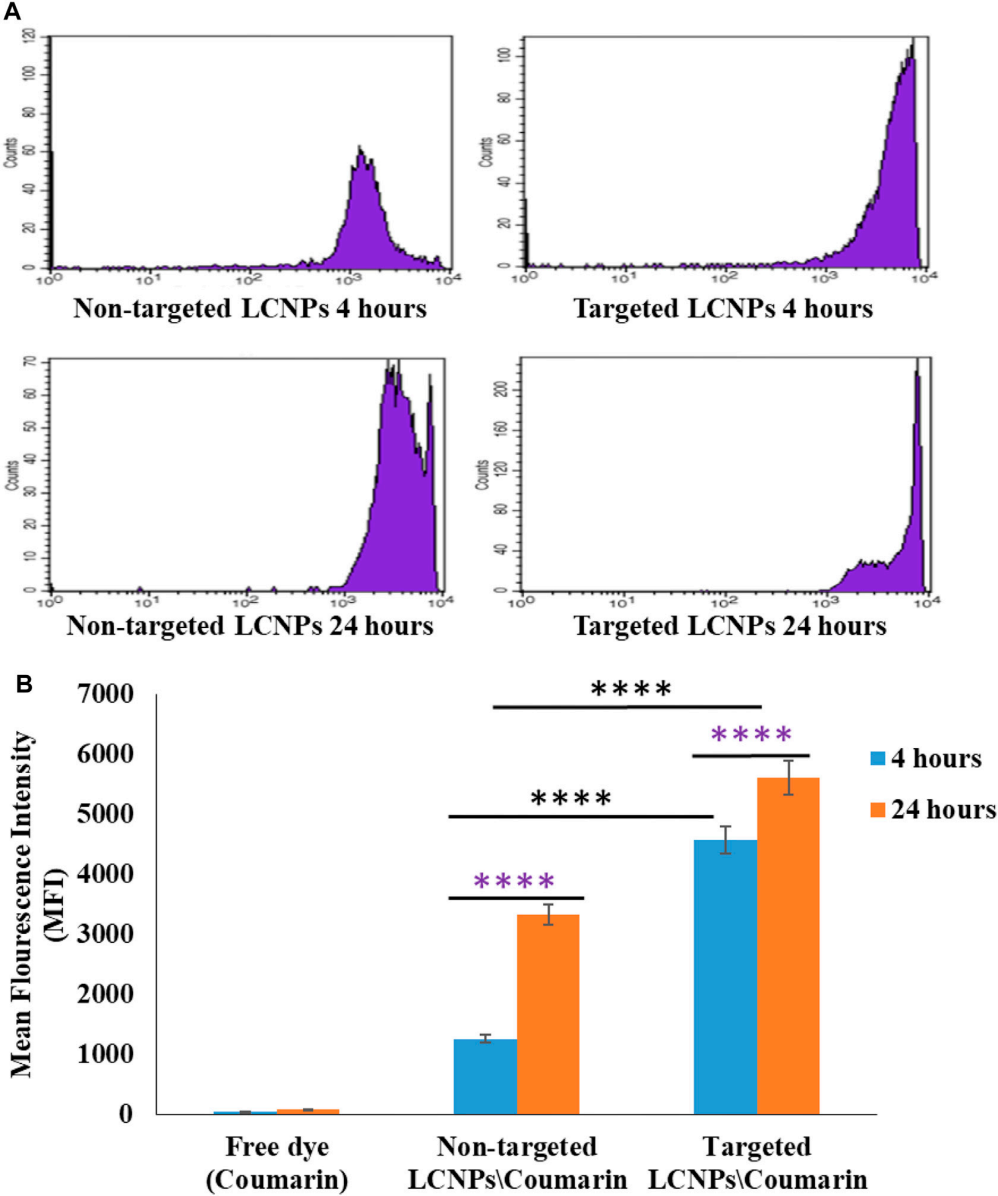
**(A)** Flow cytometry histogram profiles of MCF-7 breast cancer cell line treated with coumarin-loaded non-targeted and Lf-targeted coumarin-labeled LCNPs for 4 and 24 h at 37°C. **(B)** Quantification of cellular level of mean fluorescence intensity in MCF-7 cells after 4- and 24-h incubation with free coumarin dye, non-targeted, and Lf-targeted coumarin-labeled LCNPs after 4 and 24 h at 37°C (*n* = 3, ****p < 0.0001).

Quantitative evaluation of the intracellular uptake efficiency of LCNPs was done by flow cytometry as demonstrated in Figures 8A,B. The cellular internalizations of untargeted and Lf-targeted LCNPs were evaluated as a function of mean fluorescence intensity (MFI) calculated from flow cytometry data. The cellular levels of MFI confirmed the higher uptake efficiency with the targeted LCNPs compared to the untargeted LCNPs noticed in the confocal images. The cellular uptake performance of Lf-targeted LCNPs in MCF-7 cells could be explained by the receptor-mediated endocytosis performed by Lf receptors on MCF-7 cells. These results are in accordance with published literature, which indicate that Lf improves the efficacy of NPs against the estrogen receptor-positive breast cancer cells (Duarte et al., 2011).

## 4 Conclusion

In this study, lactoferrin-targeted self-assembled liquid crystalline nanoparticles (LCNPs) for the co-delivery of exemestane (EXE) and methotrexate (MTX) were synthesized. Lactoferrin was conjugated to the highly potent drug, methotrexate, *via* an amide bond to prevent its release in the systemic circulation and allow its controlled release in the presence of lysosomal enzymes. MTX–Lf conjugate was then deposited on the surface of EXE-loaded LCNPs by electrostatic interaction forming targeted dual drug-loaded LCNPs that can both passively and actively target breast cancer cells. The optimized formulation showed acceptable particle size (<200 nm), PDI, and good stability. Moreover, it exhibited high entrapment efficiency of EXE and acceptable conjugation efficiency of MTX, with sustained release profile for both drugs. *In vitro* cytotoxicity study on MCF-7 breast cancer cells showed that MTX/Lf\EXE\LCNPs **F4** exhibited stronger antitumor activity than the free drug combination (CI 0.242 vs. 0.342, respectively) besides demonstrating superior cellular uptake to nontargeted LCNPs. This provided evidence that targeted dual drug-loaded LCNPs could be considered a potential candidate for combined hormonal chemotherapy that can move to further *in vivo* studies to establish their efficiency in breast cancer preclinical model.

## Data Availability

The original contributions presented in the study are included in the article/Supplementary Material, further inquiries can be directed to the corresponding authors.
