# Correction: Methotrexate-lactoferrin targeted exemestane cubosomes for synergistic breast cancer therapy

**DOI:** 10.3389/fchem.2026.1883859

**Published:** 2026-06-04

**Authors:** Sarah Mokhtar, Sherine N. Khattab, Kadria A. Elkhodairy, Mohamed Teleb, Adnan A. Bekhit, Ahmed O. Elzoghby, Marwa A. Sallam

**Affiliations:** 1 Cancer Nanotechnology Research Laboratory (CNRL), Faculty of Pharmacy, Alexandria University, Alexandria, Egypt; 2 Department of Industrial Pharmacy, Faculty of Pharmacy, Alexandria University, Alexandria, Egypt; 3 Chemistry Department, Faculty of Science, Alexandria University, Alexandria, Egypt; 4 Department of Pharmaceutical Chemistry, Faculty of Pharmacy, Alexandria University, Alexandria, Egypt; 5 Pharmacy Program, Allied Health Department, College of Health and Sport Sciences, University of Bahrain, Al-Manamah, Bahrain

**Keywords:** cubosomes, exemestane, breast cancer, methotrexate, lactoferrin

There was a mistake in Figure 3 as published. The black dashed inset (the zoomed-in image on the right side of panel F) was intended to magnify a portion of the field to show particle details. However, the specific region magnified corresponds to the zone between the two dashed yellow guidelines in the original image. To avoid confusion, the corrected Figure 3 shows the same TEM image with a red box perfectly corresponding to the magnified area. The corrected Figure 3 appears below.

**FIGURE 3:**
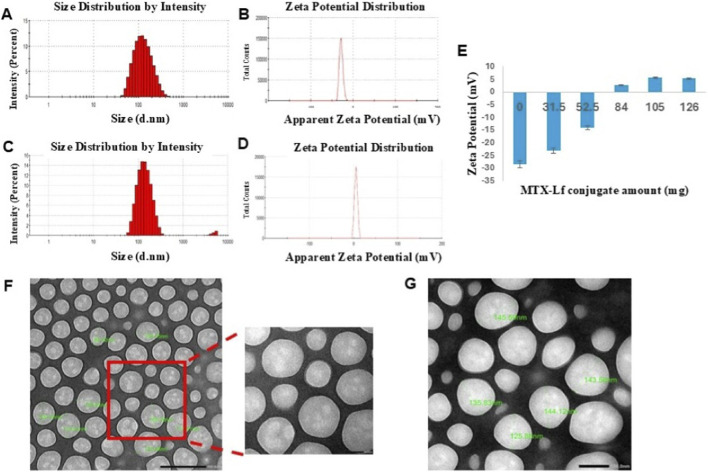
**(A)** Size distribution of EXE\LCNPs **F2**, **(B)** zeta potential of EXE\LCNPs **F2**, **(C)** size distribution of MTX/Lf\EXE\LCNPs **F4**, **(D)** zeta potential of MTX/ Lf\EXE\LCNPs **F4**, **(E)** effect of addition of MTX -Lf conjugate **F3** amounts on the zeta potential of EXE\LCNPs **F2**, **(F)** TEM of EXE\LCNPs **F2**, and **(G)** TEM of MTX/ Lf\EXE\LCNPs **F4**.

There was a mistake in Figure 4 as published. Panel B illustrating DSC thermograms for samples F2 and F3 inadvertently displayed TGA data instead of DSC data. This occurred due to unintentional oversight during retrieving data from the original folder containing both analyses for figure preparation. The corrected Figure 4 shows the accurate DSC thermograms for samples F2 and F3. Importantly, the corrected DSC data supports the same conclusions and does not affect the overall study outcomes. The corrected Figure 4 appears below.

**FIGURE 4:**
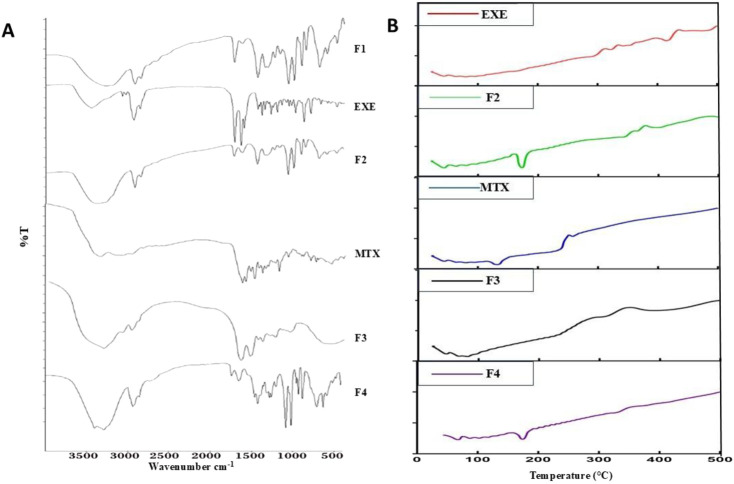
**(A)** FTIR spectra of blank LCNPs **F1**, EXE, EXE\LCNPs **F2**, MTX, MTX–Lf conjugate **F3**, and MTX/Lf\EXE\LCNPs **F4**. **(B)** DSC thermograms of EXE, EXE\LCNPs **F2**, MTX, MTX–Lf conjugate **F3**, and MTX/Lf\EXE\LCNPs **F4**.

The original article has been updated.

