# Supplementary material for: Methotrexate-Lactoferrin Targeted Exemestane Cubosomes for Synergistic Breast Cancer Therapy
Source: Front Chem. 2022 Mar 22;10:847573. doi: 10.3389/fchem.2022.847573 (PMC8980280; doi:10.3389/fchem.2022.847573)
Supplement: Supplementary file 1 [file DataSheet1.pdf]

Supporting Information

Content:

1. **Experimental**
   1.1. Materials
   1.2. Physicochemical characterization of dual drug loaded lactoferrin targeted LCNPs (MTX/Lf\EXE\LCNPs) **F4**
      1.2.1. Drug content:
      HPLC Assay of Exemestane (EXE)
      HPLC Assay of Methotrexate (MTX)
      Calculation of encapsulation and conjugation efficiency:
      1.2.2. Particle size and zeta potential
      1.2.3. *In-vitro* drug release
      1.2.4. Morphological analysis
      1.2.5. Differential scanning calorimetry (DSC)
      1.2.6. Fourier Transform Infrared (FTIR) spectroscopy
      1.2.7. Nuclear magnetic resonance (^{1}H-NMR)
      1.2.8. MALDI-TOF/TOF
      1.2.9. Physical stability
      1.2.10. *In-vitro* serum stability
   1.3. *In vitro* cytotoxicity study:
      1.3.1. Cell culture
      1.3.2. Cytotoxicity assay
      1.3.3. *In-vitro* cellular uptake:
         1.3.3.1. Confocal Microscopy Study
         1.3.3.2. Flow cytometry Study
   1.4. Statistics
2. **Results and Discussion**
   2.1. Physicochemical characterization of dual drug loaded lactoferrin targeted LCNPs (MTX/Lf\EXE\LCNPs) **F4**
      2.1.1. *In vitro* drug release
      2.1.2. Physical stability

   2.2. ***In vitro* cytotoxicity study and *In-vitro* cellular uptake**
      2.2.1. Cytotoxicity assay
      2.2.2. Flowcytometry study

1. **Experimental:**

1.1. Materials:

Exemestane (EXE) was purchased from Hong Kong Guokang BioTechnology Co., Ltd. (Shaanxi, China), Methotrexate (MTX) was purchased from Tokyo Chemical Industry (Japan), Glyceryl monooleate (GMO; Peceol®) was kindly provided by Gattefosse (France), Poloxamer-407 (P407) was obtained from (Fluka, Germany), Lactoferrin (LF) was kindly obtained from Westland milk products, 1-Ethyl-3-(3-dimethylamino) propylcarbodiimide hydrochloride (EDC.HCl) were purchased from Sigma–Aldrich (St. Louis, USA), Absolute ethanol, methanol, potassium phosphate dibasic (KH_2PO_4), and orthophosphoric acid were purchased from ADWIC, El-Nasr Pharmaceutical Chemicals Co. (Cairo, Egypt), Methanol HPLC grade was acquired from JT Baker (Phillipsburg, NJ, USA), Potassium salt of ethyl (hydroxyimino) cyanoacetate (K-Oxyma) was prepared in our Lab, Human breast adenocarcinoma cell line MCF-7 cell line was purchased from the American Type Culture Collection (ATCC), USA, Dulbecco's modified eagle medium (DMEM); composed of inorganic salts, amino acids vitamins and others, Fetal Bovine Serum (FBS), 3-(4,5dimethylthiazol-2-yl)-2,5-diphenyltetrazolium bromide (MTT), bovine serum albumin, Haematoxylin solution, Eosin solution and Canada balsam were purchased from Sigma–Aldrich (St. Louis, USA), Heparinized tubes (BD Vacutainer® Lithium Heparin 37 USP unit, BD Franklin Lakes, NJ), 2-(4-Ethoxyphenyl)-6-[6-(4-methylpiperazin-1-yl)-1*H*-benzimidazol-2-yl]-1*H*-benzimidazole (Hoechst) was purchased from Thermo-Fisher (Waltham, MA, USA), DPX – mounting medium was purchased from Loba chemie (Mumbai, India).

1.2. Physicochemical characterization of dual drug loaded lactoferrin targeted LCNPs (MTX/Lf\EXE\LCNPs) F4:

1.2.1. Drug content:

HPLC Assay of Exemestane (EXE):

Quantification of exemestane was performed using the HPLC method developed and validated in our laboratory. An Agilent 1260 Infinity HPLC system equipped with a quaternary pump, an autosampler, DAD (Diode Array Detector), and an Agilent Chemstation data processing system was used for the analysis. The HPLC analysis was carried out with an Agilent Zorbax Eclipse XDB C_{18} reversed-phase column (250×4.6 mm, 5 µm particle size) maintained at room temperature. For exemestane chromatographic elution, the injection volume was 10 µL. The mobile phase consisted of [80%:20%] methanol: water. Total run time was 6 minutes pumped at flow rate 1 mL/min, exemestane was detected at 246 nm, retention time 5.6 min.

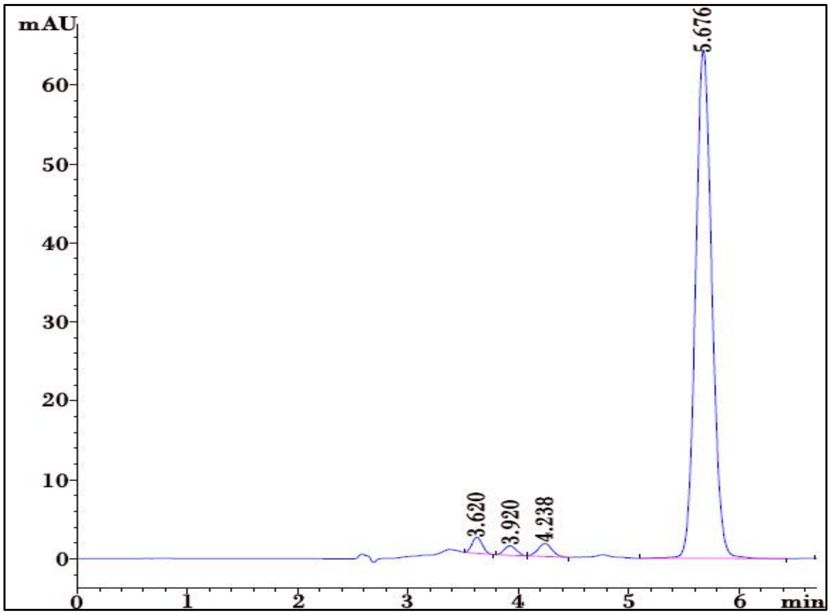

Figure 1: Exemestane HPLC chromatogram

HPLC Assay of Methotrexate (MTX):

Quantification of pemetrexed was performed using the HPLC method developed and validated in our laboratory. An Agilent 1260 Infinity HPLC system equipped with a quaternary pump, an auto sampler, vacuum degasser, DAD (Diode Array Detector), and an Agilent Chemstation data processing system was used. The HPLC analysis was carried out with an Inertsil® ODS-3 reversed-phase column (250 × 4.6 mm, 5 µm, GL Sciences Inc.). The column was maintained at room temperature. For methotrexate chromatographic elution, the injection volume was 5 µL. The mobile phase consisted of [30%:70%] methanol:Phosphate buffer solution (pH 3) adjusted using orthophosphoric acid (0.1 M). Total run time was 7 minutes pumped at flow rate 1.2 mL/min., MTX was detected at 303 nm with a retention time of 5.651min.

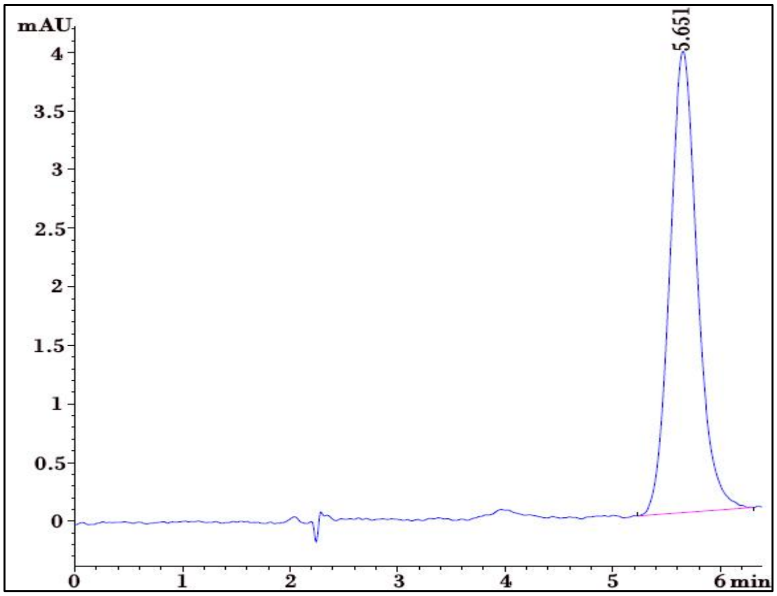

Figure 2: Methotrexate HPLC chromatogram

Calculation of encapsulation and conjugation efficiency:

Encapsulation efficiency (EE %) was performed using ultrafiltration technique (Elgindy et al., 2016). EXE-loaded LCNPs **F2** (1 mL) was placed in centrifugal ultrafilter (Vivaspin® 6 [Vivaproducts, MA, USA], molecular weight cutoff [MWCO]: 100,000). The ultrafilters were then centrifuged at 5000 rpm for 30 min. The amount of unentrapped EXE separated in the filtrate was determined by HPLC at λ_{max} 246 nm. The loaded exemestane in the LCNPs was calculated as difference between the total amount of drug used and the amount present in the filtrate (free drug) analysed by HPLC as previously described. The entrapment efficiency (EE) of EXE in LCNPs was calculated according to the following equation:

$$\% \text{ EE} = \frac{\text{Total drug} - \text{Free drug}}{\text{Total drug}} \times 100 \qquad \text{Eq. 1}$$

Where % EE is the percentage encapsulation efficiency. Total drug = wt_{total}, is the total amount of drug used and free drug = wt_{free} is the amount of free drug.

Conjugation Efficiency (CE%) of MTX:

The percent of MTX & targeting agent (Lf) conjugated was calculated indirectly by estimating the un-conjugated drug during equilibrium dialysis method using HPLC. The conjugation efficiency percentage (CE%) was calculated using the following equation,

$$\% \text{ CE} = \frac{\text{Initial drug} - \text{Free drug}}{\text{Initial drug}} \times 100 \qquad \text{Eq. 2}$$

Where % CE is the percentage conjugation efficiency. Total drug = wt_{total}, is the total amount of drug used and free drug = wt_{free} is the amount of free drug.

Each result is the mean of at least three separate experiments.

1.2.2. Particle size and zeta potential:

Particle size (PS) of LCNPs was measured by photon correlation spectroscopy (PCS) with a NanoZS/ZEN3600 Zetasizer. The PS was measured with the non-invasive backscattering technology at a detection angle of 173° after dilution with purified water to an appropriate concentration (Elzoghby et al., 2013). All of the DLS measurements were performed at 25.0±0.1°C for three repeated measurements. The zeta potential of the prepared NPs each diluted NPs suspension (1 mL) was put in a universal folded capillary cell equipped with platinum electrodes and was estimated following the same method of that used for measuring LCNPs zeta potential.

1.2.3. *In-vitro* drug release:

In-vitro exemestane and methotrexate release from self-assembled liquid crystalline nanoparticles against *in-vitro* release of each free drug suspension were performed using the dialysis bag technique (El-Far et al., 2018a). An equivalent to 2 mg exemestane and methotrexate of these dispersions was sealed in dialysis bags (12–14 kDa MWCO VISKING dialysis tubing, SERVA, Germany) and then immersed into a beaker containing 200 mL of sodium lauryl sulphate/ phosphate buffer saline (PBS) pH 7.4 (0.5 % w/v) to ensure sink conditions. The experiments were maintained at 37 °C and the system was continuously shaken at 100 rpm in a thermostatically controlled shaking water bath. At designated time intervals, 2 mL of the release medium was withdrawn and replaced by an equal volume of fresh prewarmed release medium. The same experiment was repeated but the dialysis bags were suspended in 200 mL of pH 5.5 sodium lauryl sulphate/ phosphate buffered saline (0.5 % w/v) (adjusted to maintain sink conditions and maintained at 37ºC in a shaking water bath at 100 rpm). All samples were run in triplicates and filtered through a 0.45 μm membrane filter, and the amount of EXE and MTX released was analyzed by HPLC. The cumulative amount of drug release over the time period was plotted against the time (h).

1.2.4. Morphological analysis:

The structure of the prepared targeted dual drug loaded LCNPs dispersion **F4** was examined using transmission electron microscopy (TEM) at an acceleration voltage of 80 kV. Prior to

TEM examination, the dispersion was diluted with distilled water and sonicated for 5 minutes. A drop of the diluted dispersion was placed on a copper-coated grid leaving a thin film. Sample was subsequently negatively stained by aqueous solution of uranyl acetate (1%w/v), followed by air-drying (Freag et al., 2016).

1.2.5. Differential scanning calorimetry (DSC):

DSC thermograms were recorded for EXE, MTX, EXE-loaded LCNPs **F2,** MTX-Lf conjugate **F3** and targeted dual drug loaded LCNPs **F4** using a differential Scanning Calorimeter by LINSEIS STA PT1000 thermal analyzer. Each sample (5 mg) was weighed exactly, put onto flat-bottomed aluminum pan and scanned in between 20-500 °C with a constant heating rate of 10°C/min in presence of air atmosphere (flow rate 20 mL/min) (Ali et al., 2020).

1.2.6. Fourier Transform Infrared (FTIR) spectroscopy:

The Fourier Transform Infrared (FTIR) spectra of EXE, MTX, blank LCNPs **F1**, EXE-loaded LCNPs **F2**, MTX-Lf conjugate **F3** and targeted dual drug loaded LCNPs **F4** were recorded using FTIR spectrometer. Samples were finely ground with infrared grade dry potassium bromide then pressed into pellets. The spectra were recorded in the range of 4000 to 450 cm^{-1} at ambient temperature (Elgindy et al., 2011).

1.2.7. Nuclear magnetic resonance (^{1}H-NMR):

To confirm the chemical coupling of MTX and Lf, nuclear magnetic resonance (NMR) spectrum (^{1}H-NMR) of MTX-Lf conjugate **F3** was recorded on JEOL 500 MHz spectrometers at ambient temperature. Chemical shifts are reported in parts per million (ppm) and are referenced relative to residual solvent (e.g. D_2O at δ 4.79 ppm).

1.2.8. MALDI TOF/TOF:

Exact mass measurement of MTX-Lf conjugate was carried out using a Bruker Daltonics Ultraflex MALDI TOF/TOF Mass Spectrometer in a solvent mixture of acetonitrile: 0.1% aqueous TFA (30/70).

1.2.9. Physical stability:

The physical stability of the final formulation **F4** was monitored according to terms of time and temperature of storage. Therefore, aliquots of non-diluted targeted dual drug loaded LCNPs **F4**

were placed in sealed tubes in a refrigerator at 4-6°C for storage. PS, PDI and zeta potential of the LCNPs were monitored at different time points for a period of 3 months (Fang et al., 2012).

1.2.10. *In-vitro* serum stability:

In order to mimic blood circulation conditions, targeted dual loaded formulation **F4** was incubated in a shaking water bath at 37°C under mild stirring with an equal volume of 10% w/v FBS for 6 h. At each time interval (0, 1, 2,3, 4 and 6 h), 50 µL of the mixture was withdrawn then diluted in distilled water (1:50 v/v) to be assessed for their PS and PDI, using dynamic light scattering (DLS) technique (Wolfram et al., 2014;El-Far et al., 2018b).

1.3. *In vitro* cytotoxicity study:

1.3.1. Cell culture:

The cell culture experiments were carried out on human breast adenocarcinoma MCF-7 cell line. The cells were maintained in Dulbecco's modified eagle medium (DMEM) containing 10% FBS in a CO_2 incubator (5% CO_2 at 37°C). Cells were subcultured for one week before assay as previously reported (Ungaro et al., 2012a).

1.3.2. Cytotoxicity assay:

The cells were maintained in Dulbecco's modified eagle medium (DMEM) containing 10% fetal bovine serum (FBS) in a CO_2 incubator (5% CO_2 at 37°C). Cells were seeded at a density of 5×10^3/well in a 96-well plate containing 100 µL of DMEM enriched and allowed to adhere to the plate overnight. The cytotoxicity of the the free drugs, blank LCNPs **F1**, Lf-targeted LCNPs, targeted dual drug loaded LCNPs **F4** on MCF-7 breast cancer cells was assessed by the MTT assay. Therefore, the medium was replaced by fresh medium containing different concentrations of the drugs either as a solution or the prepared LCNPs and incubated for another 24 h. A stock solution of EXE, MTX and combination of both were prepared individually in water using concentration of 1 mg/mL and stored at -70ºC. The culture medium was then replaced with 100 µL of MTT solution (0.5 mg/mL in DMEM) then incubated for further 4 h at 37°C in the dark. After removal of MTT solution by centrifugation at 2000 rpm for 10 min, 100 µL of DMSO were added to the wells to dissolve MTT-formazan crystals formed after internalization of MTT by live cells and maintained in agitation for 15 min. Absorbance of the converted dye was measured at a wavelength of 570 nm with background subtraction at 690 nm using a microplate

reader (Model 550, Bio-Rad, USA) (Ungaro et al., 2012b). The relative cell viability was expressed as a percentage of the untreated control wells. The inhibitory concentration (IC_{50}) values were determined using Origin 8.0 (Origin Lab, Northampton, MA) according to the fitted data.

The percentage of cell viability related to control cells incubated with culture medium only was determined by the following equation:

$$\%Cell\ viability\ =\ [A(test)\ /\ A(control)]\ x\ 100 \qquad \text{Eq. 4}$$

Where A (test) is the absorbance obtained from the test sample and A (control) is the absorbance obtained from untreated cells (incubated with medium only). The latter reading was assumed to correspond to 100% cell viability. The MTT assay for each concentration was performed in triplicate. The 50% cell cytotoxic concentration (IC_{50}), the concentration required to kill 50% of the cells, was estimated.

1.3.3. *In-vitro* cellular uptake:

MCF-7 cells were seeded on glass coverslips placed in six-well culture plates overnight at a density of 1.8×10^5 cells. The medium was then replaced with fresh medium adjusted to pH 7.4, which mixed with the prepared nanoparticles. After incubation, the medium was removed, and the cells were washed three times with cold PBS to terminate the cell uptake. After that, the cells were fixed with 4% paraformaldehyde solution and then the cover slips were then mounted onto microscope slides using 2-(4-ethoxyphenyl)-6-[6-(4-methylpiperazin-1-yl)-1*H*-benzimidazol-2-yl]-1*H*-benzimidazole (Hoechst) containing (DPX) as mounting medium.

The regular DMEM was applied as the blank control. All the tests were performed in triplicate.

1.3.4. Confocal Microscopy Study:

Imaging analysis was performed via confocal laser scanning microscopy using the following filter set; excitation wavelength: 488 nm, and emission wavelength 530 nm.

1.3.5. Flow cytometry Study:

Cellular uptake was quantitatively analyzed by flow cytometry. The collected cells were analyzed by a Beckman Coulter EPICS XL flow cytometer (Beckman Coulter, Fullerton, CA, USA).

1.4. Statistics:

For all in- vitro characterization, all measurements were carried out in triplicate and values are presented as the mean ± S.D. For comparison of mean values between groups, Paired t-test, Analysis of Variance test (ANOVA), Tukey's Multiple Comparison test and Fisher exact test were used. The difference was considered significant when P-values < 0.05.

2. Results and Discussion:

2.1. Physicochemical characterization of dual drug loaded lactoferrin targeted LCNPs F4 (MTX/Lf\EXE\LCNPs):

2.1.1 *In vitro* drug release

Table 1: *In vitro* release study of free EXE, free MTX, EXE\LCNPs **F2** and MTX/Lf\EXE\LCNPs **F4** (n=3):

% Drug release					
Time (h)	Free Exemestane %	EXE loaded LCNPs **F2** %	EXE from Targeted dual loaded LCNPs **F4** %	Free Methotrexate %	MTX from Targeted dual loaded LCNPs **F4** %
0	0	0	0	0	0
0.5	18.1±0.15	10.5±0.5	10±0.19	34.8±0.39	0.005±0.0002
1	42.3±1.3	21±0.98	18.8±0.57	45.2±1.26	0.006±0.00012
2	75.2±1.9	36.8±1.2	34.5±1.4	64.6±1.31	0.009±0.00019
4	81.9±2.25	52.6±1.5	45.6±1.68	80.1±2.1	0.013±0.0002
6	85.1±2.83	63.15±1.72	58.7±1.96	82.6±2.75	0.014±0.00023
8	91.8±1.67	68.4±1.98	62.7±2.08	85±2.36	0.015±0.00018
12	95.9±1.92	70±1.79	67.1±2.27	92±1.96	0.016±0.00021
24	98.1±2.14	73.6±2.04	69.8±1.97	96.9±2.41	0.0161±0.00019

48	100±2.74	75±2.54	70±2.01	100±1.87	0.0161±0.00023

2.1.2 Physical stability:

Table 2: Effect of three-month storage on dual drug loaded targeted LCNPs **F4** on the PS, PDI, zeta potential (n=3):

	Particle size (nm)		PDI		Zeta potential (mV)	
	Before	After	Before	After	Before	After
Three-month storage	143.6±3.24	170±4.16	0.18	0.322	+5.64	+3.5

2.2. *In vitro* cytotoxicity study and *In-vitro* cellular uptake

2.2.1. Cytotoxicity assay:

Table 3: Cytotoxicity analysis of free EXE, free MTX, free EXE/MTX and the dual drug loaded targeted LCNPs **F4** on MCF-7 breast cancer cell line after 24 h (n=3).

	% Cell viability			
Conc. (µg\mL)	Exemestane	Methotrexate	Free combination (EXE+MTX)	Targeted EXE+MTX loaded LCNPs **F4**
0.39	58.68±2.78	43.39±2.41	41.94±2.15	37.65±1.71
1.56	51.93±3.2	37.83±2.25	37.12±2.26	30.26±2.22
6.25	45.33±2.64	28.84±1.74	29.57±1.89	24.36±1.78
25	37.14±2.48	20.45±1.11	24.45±1.21	18.22±0.75
100	30.23±2.37	9.53±0.62	13.27±0.43	7.76±0.58

Table 4: IC_{50} values of free drugs compared to the prepared dual drug-loaded LCNPs **F4** on MCF-7 breast cancer cells at the concentration of 0.39-100 µg/ml after 24 h.

	IC_{50} µg\mL
Exemestane	2.199±0.059
Methotrexate	0.302±0.005
Free EXE+MTX	0.182±0.003
Targeted dual drug loaded LCNPs **F4**	0.129±0.001

2.2.2. Flow cytometry study:

Table 5: Quantification of cellular level of mean fluorescence intensity in MCF-7 cells after 4- and 24-hours incubation with free coumarin dye, non-targeted and targeted coumarin labeled LCNPs at 37 °C (n=3).

	Mean fluorescence intensity (arbitrary units)	
	4 hours	24 hours
Free dye (coumarin)	44.91±1.58	87.38±2.02
Untargeted LCNPs\coumarin	1263.46±39	3337.62±56
Targeted LCNPs\ coumarin	4572.53±67	5623.41±72

References:

Ali, O.M., Bekhit, A.A., Khattab, S.N., Helmy, M.W., Abdel-Ghany, Y.S., Teleb, M., and Elzoghby, A.O. (2020). Synthesis of lactoferrin mesoporous silica nanoparticles for

pemetrexed/ellagic acid synergistic breast cancer therapy. *Colloids and Surfaces B: Biointerfaces* 188**,** 110824.
El-Far, S.W., Helmy, M.W., Khattab, S.N., Bekhit, A.A., Hussein, A.A., and Elzoghby, A.O. (2018a). Folate conjugated vs PEGylated phytosomal casein nanocarriers for codelivery of fungal-and herbal-derived anticancer drugs. *Nanomedicine* 13**,** 1463-1480.
El-Far, S.W., Helmy, M.W., Khattab, S.N., Bekhit, A.A., Hussein, A.A., and Elzoghby, A.O. (2018b). Phytosomal bilayer-enveloped casein micelles for codelivery of monascus yellow pigments and resveratrol to breast cancer. *Nanomedicine* 13**,** 481-499.
Elgindy, N., Elkhodairy, K., Molokhia, A., and Elzoghby, A. (2011). Biopolymeric microparticles combined with lyophilized monophase dispersions for controlled flutamide release. *International journal of pharmaceutics* 411**,** 113-120.
Elgindy, N.A., Mehanna, M.M., and Mohyeldin, S.M. (2016). Self-assembled nano-architecture liquid crystalline particles as a promising carrier for progesterone transdermal delivery. *International journal of pharmaceutics* 501**,** 167-179.
Elzoghby, A., Helmy, M., Samy, W., and Elgindy, N. (2013). Micellar Delivery of Flutamide Via Milk Protein Nanovehicles Enhances its Anti-Tumor Efficacy in Androgen-Dependent Prostate Cancer Rat Model. *Pharmaceutical Research* 30**,** 2654-2663.
Fang, M., Jin, Y., Bao, W., Gao, H., Xu, M., Wang, D., Wang, X., Yao, P., and Liu, L. (2012). In vitro characterization and in vivo evaluation of nanostructured lipid curcumin carriers for intragastric administration. *Int J Nanomedicine* 7**,** 5395-5404.
Freag, M.S., Elnaggar, Y.S., Abdelmonsif, D.A., and Abdallah, O.Y. (2016). Layer-by-layer-coated lyotropic liquid crystalline nanoparticles for active tumor targeting of rapamycin. *Nanomedicine* 11**,** 2975-2996.
Ungaro, F., D'angelo, I., Coletta, C., D'emmanuele Di Villa Bianca, R., Sorrentino, R., Perfetto, B., Tufano, M.A., Miro, A., La Rotonda, M.I., and Quaglia, F. (2012a). Dry powders based on PLGA nanoparticles for pulmonary delivery of antibiotics: modulation of encapsulation efficiency, release rate and lung deposition pattern by hydrophilic polymers. *J Control Release* 157**,** 149-159.
Ungaro, F., D'angelo, I., Coletta, C., Di Villa Bianca, R.D.E., Sorrentino, R., Perfetto, B., Tufano, M.A., Miro, A., La Rotonda, M.I., and Quaglia, F. (2012b). Dry powders based on PLGA nanoparticles for pulmonary delivery of antibiotics: modulation of encapsulation efficiency, release rate and lung deposition pattern by hydrophilic polymers. *Journal of controlled release* 157**,** 149-159.
Wolfram, J., Suri, K., Huang, Y., Molinaro, R., Borsoi, C., Scott, B., Boom, K., Paolino, D., Fresta, M., and Wang, J. (2014). Evaluation of anticancer activity of celastrol liposomes in prostate cancer cells. *Journal of microencapsulation* 31**,** 501-507.
